# The Effect of Verbal and Iconic Messages in the Promotion of High-Quality Mountain Cheese: A Non-Hypothetical BDM Approach

**DOI:** 10.3390/nu13093063

**Published:** 2021-08-31

**Authors:** Maria Elena Marescotti, Mario Amato, Eugenio Demartini, Francesco La Barbera, Fabio Verneau, Anna Gaviglio

**Affiliations:** 1 Department of Veterinary Science for Health, Animal Production and Food Safety, University of Milano, Via dell’Università 6, 26900 Lodi, Italy; maria.marescotti@unimi.it (M.E.M.); eugenio.demartini@unimi.it (E.D.); anna.gaviglio@unimi.it (A.G.); 2 Department of Political Sciences, University of Napoli “Federico II”, Via Rodinò 22a, 80138 Napoli, Italy; francesco.labarbera@unina.it (F.L.B.); verneau@unina.it (F.V.)

**Keywords:** mountain livestock farming, grass-fed, pastures, mountain food products, experimental auctions, willingness-to-pay, animal welfare, Italy

## Abstract

To ensure the sustainable development of mountain livestock farming, the adequate remuneration of high-quality dairy products is fundamental. In this sense, communication strategies aimed at promoting mountain products and ensure better positioning and higher market prices are fundamental. The present research seeks to expand the literature regarding consumers’ willingness to pay for mountain foods by using an online real auction experiment aimed at evaluating the premium price that consumers are willing to pay for summer over winter mountain cheese, depending on the information provided concerning the taste anticipation or animal welfare. The results showed an overall small premium price given to the higher quality summer cheese; this could be, partially, due to a generally low degree of consumer knowledge about mountain dairy farming. With reference to communication strategies, the results provide evidence about the effectiveness of the rational messages founded upon sensorial characteristics and the anticipated taste of cheese. In addition, this study explored that adding a logo had no main effect on the price premium participants were willing to pay. This may be due to the fact that logos and claims, having a lower information content, are more indicated to lead the choice of consumers with a higher level of awareness. In the conclusion section, policy and agribusiness implications of the findings are provided.

## 1. Introduction

The Alps are the most densely populated mountainous area of the world, however, while the populations of coastal touristic mountains and semi-urbanized foothills are growing, most of the internal valleys are suffering a slow but apparently inexorable depopulation which endangers the long-term survival of alpine communities and their economic activities [1,2]

In this sense, alpine livestock farming is showing one of the most alarming shifts. Farming of ruminants fed on pastures for production of milk and cheese drove the mountain economies for centuries; however, the new generations of residents have abandoned these traditional activities [2,3,4,5,6]. The reasons behind this defection are mainly related to the natural difficulties of mountain agriculture, due to the existence of natural and technological constraints, such as a shorter growing season, the presence of slopes, which impede the use of machinery, and poor mobile network coverage [7,8,9,10,11]. 

These aspects make alpine livestock farming suffer a lack of profitability due to a lower land and labor productivity when compared to lowland agriculture [7,11,12]. In this context, the ecosystem services of extensive livestock farming must be mentioned: such as the conservation of biodiversity, balance between native and invasive species, prevention of fires, maintenance of landscapes, conservation of the soil by reducing the risk of erosion, conservation of the organic wealth of the soil, less pollution, and better management of water resources. Livestock farming constitutes a mitigation strategy in the fight against climate change due to the carbon sequestration of pastures, contributes to job creation and to the structuring of the rural environment, conserves the landscape, conserves socio-cultural resources, and so on [13].

Given the handicaps faced by mountain farmers, the European Union supports their activities with the Common Agricultural Policy schemes [14]. These contributions have only partially hindered alpine farming abandonment, which implies the loss of livestock genetic resources and of typical dairy products and the decline of unique semi-anthropic ecosystems, such as pastures, grasslands, and terraces, with a great environmental and aesthetic value [7,15,16,17]. While the public payments represent a fundamental tool to support the maintenance of agricultural activities in this area in the short-term, efficient solutions are still required.

The recent literature proposes the valorization of mountain food products as a feasible way to improve economic viability and ensure the sustainable development of local alpine farming in the long-term [18,19,20,21]. In fact, modern consumers are interested in sustainable, traditional, and local foods [22,23,24,25]. In this respect, alpine livestock farming presents some promising characteristics. For instance, during summer, the availability of pastures allows farmers to produce milk and cheeses with unique features. 

Pasture-based feeding is associated with products of higher organoleptic and nutritional quality compared to those produced by animals fed with a conventional concentrate-forage diet [26,27,28,29]. In fact, as reported by several authors [13,30,31,32,33], pasture-based milk and cheese contain higher concentrations of polyunsaturated fatty acids (PUFAs), such as α-linolenic acid, vaccenic acid, conjugated linoleic acid (CLA), and fat-soluble antioxidants, which help in improving the fatty acid composition and the antioxidant potential of the human diet [34,35,36,37,38], thus, lowering the risk of cancer and cardiovascular diseases [39,40], and confer typical organoleptic properties to cheeses [41,42,43,44,45]. 

Secondly, summer pastures provide dairy cows with more space and consent them to follow their natural behaviors, which implies a higher level of animal welfare [46,47]. On the other hand, it is worth emphasizing that the nutritional and organoleptic quality of pasture-based milk and cheese might not be assured during winter season, due to the lack of fresh grass and the need to shelter the cows in stables. 

Another aspect to highlight is that pastures do not guarantee higher animal welfare per se; on the contrary, some mountain farmers fail in providing dairy cows with adequate welfare in the summer season because of mis-practices [46,47]. Nonetheless, if farmers adequately manage the herds, grassland-based milk production systems can be considered as sustainable, safe, and delivering high-quality products [48].

With these factors considered, some authors studied the consumer willingness to pay a price premium for mountain foods. Research by Sajuàn and Khliji [49] found that mountain beef is appreciated by a niche of consumers; however, the average consumer is only slightly willing to pay more for mountain products compared to conventional ones. More recently, Nam et al. [50] and Mazzocchi et al. [51] found higher willingness to pay for mountain milk and cheese respectively in consumers with higher awareness of animal welfare and agricultural sustainability issues. These results suggest that only specific clusters of consumers would pay more for this characteristic.

Even if consumers appreciate mountain dairy products as unique, tasty, and sustainable food, the problem raises in the real context, where consumers do not pay a higher price because multiple factors affect their final purchasing choices. A part of individual characteristics, in fact, many external variables may influence individual attitudes and/or nudge real food choices [52,53]. One of the most interesting issue relates to the effect of information on perception of food. 

For example, in recent research, Verneau et al. [54] measured the effect of information framing and implicit associations in consuming insects, while Demartini et al. [55] measured the effect of rational informative health message and self-reference treatment on consumers’ preferences for functional foods, proving that effective strategies can be designed to improve consumers’ attitudes toward foods. 

Finally, it is worth noting that, in many cases, people do not behave as they declare in a survey. Thus, in a hypothetical scenario, they tend to underestimate the costs of their choices and/or overestimate their willingness to pay for high quality foods [56,57,58,59]. As a result, the real market premium price for high-quality mountain dairy products does not always compensate mountain farmers for the low productivity of grassland-based farms [51,60].

Therefore, there is a pressing need to design specific interventions to promote mountain products to ensure better product positioning and higher market prices. The present study attempts to fill this gap and contribute to the literature by proposing an online real auction experiment aimed at evaluating the real premium price that consumers are willing to pay for winter and summer mountain cheese depending on the information provided. 

The main goal is to compare different communication strategies and messages that guarantee the highest price for the summer cheese production, obtained through pasture-based feeding and associated with products of higher organoleptic and nutritional quality. Specifically, the effect of two different types of information delivered by rational messages was tested in the study. The first one related to the higher animal welfare provided by summer pastures, the second one related to the unique sensory characteristics of the summer cheese. 

Furthermore, the effect of two logos on the living and feeding conditions of dairy cows during summer was tested. Specifically, the first logo referred to the fact that the cows are reared “free-range” (translated from the Italian: “vivono all’aria aperta”) and are “grass-fed” (translated from the Italian “erba fresca”). The paper contributes to the literature in at least three ways. First, we are not aware of any research that tested the effect of information treatments on consumers’ preferences for sustainable mountain food. Secondly, we measured consumers’ willingness to pay in a real purchase scenario. Finally, we used a Becker–DeGroot–Marschak (BDM) [61] online auction to collect the data.

## 2. Aims and Hypothesis

The current experiment investigated the effects of different types of communication on participants’ willingness to pay (WTP) for mountain summer cheese (SMC) versus winter cheese (WMC). To achieve this goal, we first investigated whether individuals were ready to pay a premium price for SMC compared to WMC. 

Secondly, we investigated whether the participants’ premium price WTP was affected by a rational message based on the possible positive consequences of summer pastures on animal welfare (*info-ANW*) versus the products’ taste (*info-TAS*). Third, we investigated whether adding a logo about free-range rearing (*logo-FRE*) versus grass-fed (*logo-GRF*) had an influence on the price premium that participants were willing to pay for SMC versus WMC. Finally, the interactions between the types of rational massages by the logos were assessed.

Hence, the experimental design was defined by two between-participant factors (rational message: *info-ANW* vs. *info-TAS* vs. *No info*; logo: *logo-FRE* vs. *logo-GRF* vs. *No logo*) and one within-participant factor (Cheese: *SMC* vs. *WMC*). The participants were randomly attributed to one of the nine experimental conditions.

A premium price for SMC was expected; however, only in the experimental conditions in which information about the differences between the two products was provided (*info-ANW* and *info-TAS*), whereas no premium price was expected in the control condition (*No info*), in which this information was not provided to the participants. In addition, we expected a significant and positive interaction between the two between-participant factors, namely rational message and logos, because the logo were expected to confirm the message, thus, reinforcing its effect.

## 3. Materials and Methods

The experiment was designed to compare the effect of different information treatments, both verbose and non-verbose, on the WTP for high quality mountain cheeses. To assess the participants’ willingness to pay, a BDM incentive-compatible mechanism was implemented using Veylinx software (Veylinx, Amsterdam, The Netherlands), which is an online experimental auction platform.

The online setting was preferred for many competing factors. Although experimental auctions are generally carried out in ad-hoc laboratories or in-store, the COVID-19 pandemic prompted researchers to find a viable alternative to traditional settings, without compromising the validity of the results. Furthermore, since every participant could carry out the tasks in the isolation of their houses, remotely and safely, an online setting has the benefit to minimize socially desirable behavior and the possible urge to please the experimenter [62]). Lastly, an online setting allows a much larger stratified sample to be reached compared to laboratory settings, therefore, enhancing the external validity of the results.

### 3.1. The BDM Online Auction

Despite the availability of many different mechanisms to investigate consumer preferences and the WTP for new food products or attributes, scholars have shown a strong preference for experimental auctions [59]. In particular, the most widely used tools are the *n*th price sealed bid auction, also called the Vickrey auction (VA) [63], and the Becker–DeGroot–Marschak auction (BDM) [61].

In an *n*th price sealed bid auction, each subject simultaneously submits a bid to purchase a good. The agent(s) who submits the highest bid wins the auction and receives the item but pays an amount equal to the second highest bid in the auction. In the BDM auction, a participant is asked to express a monetary value that (s)he would be willing to pay for the product. To win the product, each participant must submit a bid that truly represents the value (s)he attaches to the auctioned good. If this bid is greater than or equal to a randomly extracted binding value, the participant must buy the auctioned product at the price equal to the binding value. On the contrary, if participant’s bid is lower than the randomly extracted value, the purchase does not happen.

The choice of BDM auction was influenced by several competing factors. A BDM auction avoids competition among participants since the decision is made in isolation and the outcome has only consequences for the decision maker, whereas in a VA, a strategic interaction between the participants exists, and the outcome is also determined by the other participants’ decisions, since each participant is aware that the change in the declared bid will have consequences on the outcome. 

Furthermore, differently from a BDM auction, where the result of the auction is immediately available to participants, online Vickrey auctions are usually run among a period of time that can vary between few hours to few days [64,65], which may possibly cause a lack of involvement. Finally, the BDM auction is easy to explain and does not require a long training phase [66,67], which is an optimal feature for an online experiment that does not allow constant control by the experimenter.

### 3.2. Experimental Stimuli

#### 3.2.1. The Products

**Figure 1:**
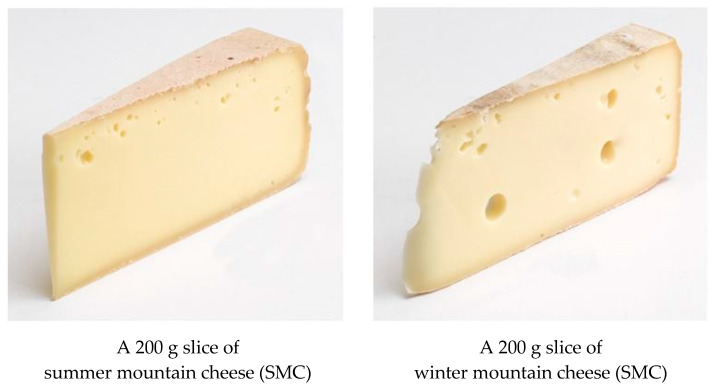
The summer and winter mountain cheeses that were auctioned.

The auctioned products used in the study were two high quality mountain cheeses, produced in the summer (SMC) or winter (WMC) season. Specifically, participants were presented with two real pictures representing a 200 g slice of SMC and WMC that were taken by a professional photographer (Figure 1). The two types of auctioned mountain cheeses were produced using whole fresh milk using the same recipe by the one farm and differentiated by the season of production. The two products are sold in real markets using different names that recall the fact that one is produced during summer and the other during winter.

#### 3.2.2. The Information Treatments

As the study aimed at testing the effects of two different informative rational messages and their interactions with two logos on consumers WTP for SMC versus WMC. A review of the literature on consumers’ attitudes toward cheese mountain products was conducted, and the results revealed that the quality aspects of animal welfare and taste played a relevant role in shaping consumers’ preferences for this type of product. Thus, these two aspects, namely animal welfare and taste, were incorporated into two different rational messages (see Appendix A) explaining the differences between high quality mountain cheese products obtained during the summer season compared to winter mountain cheese. In line with previous research, messages with similar length, structure, and complexity were employed [54,68].

A pilot-study was conducted for developing the logos. As a first step, five different logos were reviewed by experts in the field. The chosen logo was a plain and stylized image of a cow, white on blue background. Five different short claims were developed and tested on a convenience sample of 115 participants. 

Individuals’ beliefs elicited by the combination of the logo plus each of the claims were explored through a free association task [69]: participants were divided in five groups by a random draw; subsequently, they were asked to write the first five adjectives that came to mind in relation to the image shown to them (i.e., the combination of one logo and to each group). The content analysis showed a coherent set of beliefs only for two out of five combinations, namely those reporting the claims about open air rearing (*logo-FRE*) and grass-feeding (*logo-GRF*), whereas the other three combinations elicited ambivalent beliefs and were, therefore, dropped.

Thus, a logo informing that, during summer, the dairy cows are reared open air or are grass-fed was added to the summer cheese picture to recall its increased animal welfare or taste, respectively, compared to the winter cheese (see Appendix B).

### 3.3. Participants

The experiment was carried out between the end of June and the first week of July, 2020, using the Veylinx platform. Participants were recruited through a panel provider, whose members are consumers that are representative of the Italian population and were asked to participate in an online auction for cheese. They were informed that, at the end of the experiment, they would receive 15 euros as compensation for the time spent. A total of 849 participants correctly completed all the questionnaire phases; the final sample included 426 females (50.2%), with an average age of 46.4 years (SD = 15.23).

Table 1 summarizes and compares the main sociodemographic characteristics in both the collected sample and Italian population [70]; the percentages of adult education at the country level refer to the highest degree completed by the 25–64 year-old population [71]. Overall, the sample shows an adequate representation of the Italian population, despite some differences regarding the education variable, which was higher in the sample than in the Italian population. Considering that the survey was administered on the web, the discrepancy was largely expected and perfectly in line with those of other similar web-administered and self-compiled surveys [72,73,74].

### 3.4. Experimental Procedure

Upon landing on the Veylinx platform, participants were asked if they were 18 years or older and if they consumed cheese in the last two months. After these screening questions, they had the opportunity to give their informed consent for data treatment, according to the GDPR established by Regulation (UE) 2016/679. Once the consent to participate was obtained, panel members were informed that: (a) they were joining in a real auction; (b) the products in the auction were real; (c) they will be really asked to purchase (i.e., pay with their own money) the product auctioned in the case where they win the auction; and, (d) they will receive the product by mail in a few days after the auction.

Since it is of great importance that every participant carefully understood the BDM auction mechanism, they were then presented with an explanatory note, and, in order to gauge their understanding and to make sure that everything was clear, they were asked to answer a short quiz to advance to the auction [75]. The test consisted of deciding the possible outcome of a BDM auction for a touristic service. If they answered correctly, they could continue with the experiment. If they answered incorrectly, they were shown the instructions again and asked to repeat the test. If a further incorrect answer was given, the questionnaire directed them to the end, and the answer was not recorded.

**Figure 2:**
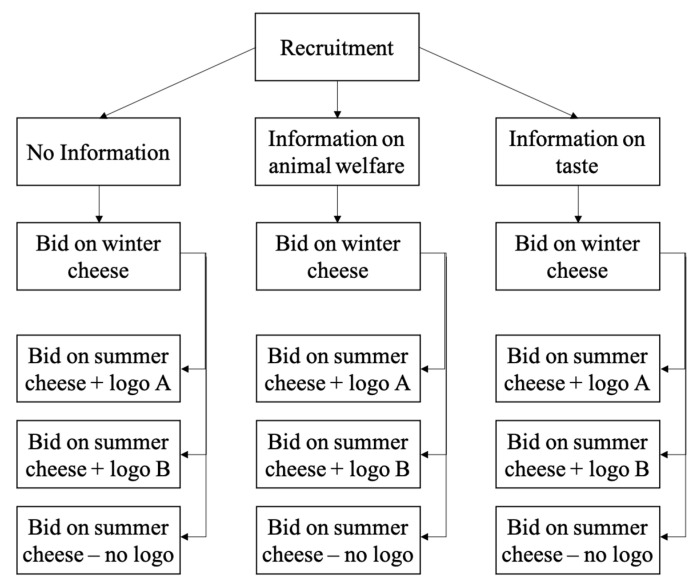
Flow of the experiment.

Participants were then randomly assigned to one of nine groups resulting from the 3 (rational message) X 3 (logo) experimental design, which is illustrated in Figure 2. As a first step, roughly equal numbers of people received one of the two messages or received no message at all (*No info* group: *n* = 281; *Info-TAS* group: *n* = 284; *Info-ANW* group: *n* = 284). Then, the proper BDM auction started, and the participants were asked to bid for a 200 g slice of mountain cheese produced during the winter season (WMC). 

Following this first auction, participants were then assigned to a further random group and were asked to place their bid for a mountain cheese produced during the summer season (SMC), which was either: (1) a 200 g slice of SMC bearing a logo indicating that the cows had been left free to graze outdoors in open air (*logo-FRE*: *n* = 288); (2) a 200 g slice of SMC bearing a logo indicating that the cheese was produced using grass-fed milk (*logo-GRF*: *n* = 280); or, (3) a 200 g slice of SMC without any further information, (*No logo*: *n* = 281). Since each participant bids on two products (SMC and WMC) some anchoring and ordering effect could occur, whereby responses to the first task influence the responses to the next one. To account for potential anchoring effects, counterbalancing in the task order was used among participants.

## 4. Results

Overall, 849 individuals correctly completed the questionnaire. As a first step, it is important to verify that the socio-demographic characteristics of the participants did not differ significantly from each other across groups. Therefore, a series of *χ*^2^ tests were ran, the non-significance of which allowed us to reject the hypothesis that the groups were different from each other with regard to gender, age, level of schooling, household members, and geographical location (*p*s < 0.10).

In line with previous studies [75,76], the rate of involvement in the market was quite high: 64% (*n* = 543) of participants declared positive prices for both WMC and SMC; this percentage rose to 78% (*n* = 660) if those individuals who expressed a positive price for at least one product were considered. Finally, 22% (*n* = 189) systematically excluded themselves from the market by declaring zero for both SMC and WMC. Noticeably, the experimental conditions related to the information message and the logo did not influence this decision (*χ*^2^ = 19.122, *p =* 0.262).

The main descriptive statistics of the bids are shown in Table 2. As expected, the distribution was positive skewed. The average price, excluding zero bids, was in line with market prices for high quality dairy products. Table 3 provides a complete overview of the mean bids across experimental conditions. Looking at the total of the participants, a very small premium price of 11 Euro cents for SMC over WMC was found. In line with the expectations, the difference in willingness to pay for SMC and WMC was qualified by the type of information received. Participants in the *info-TAS* condition showed a premium price of 42 Euro cents. While the absence of a significant premium price in the control condition (*No info*) is not surprising, unexpectedly, participants in the *info-ANW* condition also did not show a significantly higher WTP for SMC compared to WMC.

To examine the effects of the message, logo, and their interaction with the participants’ willingness to pay for winter cheese versus summer cheese, an ANOVA with a repeated measure factor, Cheese (WMC versus SMC), and two between-participant factors, Message (*Info-TAS* versus *Info-ANW* versus *No Info*) and Logo (*logo-FRE* versus *logo-GRF* versus *No logo*) was carried out. The order of cheese presentation was entered as a covariate.

There is a significant effect of the cheese on the participants’ WTP, which was overall slightly higher for summer cheese (M = 2.92) compared to winter cheese (M = 2.81), *F* (1, 838) = 4.84, *p =* 0.023, partial *η*^2^ = 0.006. Importantly, the interaction between the cheese and message was significant, *F* (2, 838) = 3.29, *p =* 0.038, partial *η*^2^ = 0.008. Simple effects analysis showed that, in the *Taste* condition, there was a significant difference between the WTP participants declared for winter cheese (M = 2.86) and the WTP they declared for summer cheese, (M = 3.28), *t* (283) = 2.08, *p =* 0.039, whereas there was no significant difference between WTP in the animal welfare condition and in the control condition (*No info*), *t*s < 1.44, *p*s = ns.

**Figure 3:**
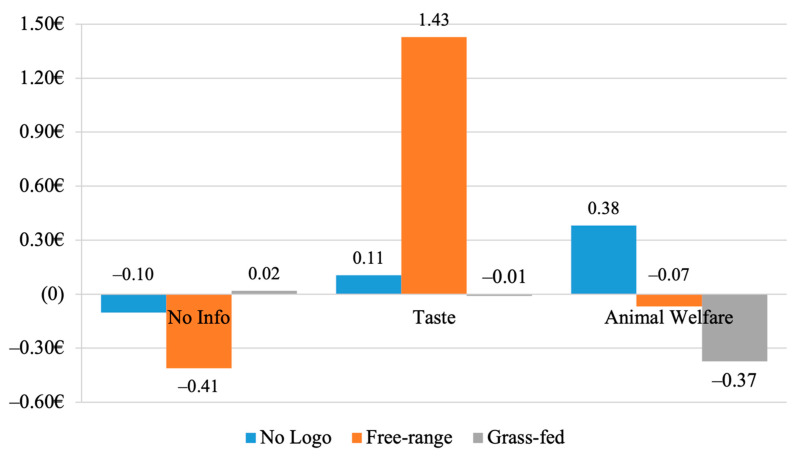
The mean difference between the WTP for SMC and WMC across message and logo conditions.

The interaction between Cheese and Logo was not significant, F < 1.34, *p =* ns. Therefore, the difference between WTP declared for summer versus winter cheese was not influenced by adding a logo on the product image. On the contrary, the three-way interaction between Cheese, Message, and Logo was significant, *F* (4, 838) = 3.08, *p =* 0.016, partial *η*^2^ = 0.014. Pair comparisons showed that the difference between WTP for summer and winter cheese for participants in the Taste condition who saw the Open-air logo was higher compared to other groups (M = 1.43), *t* (95) = 2.12, *p =* 0.037 as shown in Table 4 and Figure 3.

## 5. Discussion

The current study was conducted in the context of a rising interest concerning the promotion of mountain products, which is linked to several economic and social benefits that were reviewed in the introduction. Therefore, the main aim of the work was to explore the effects of—and interactions between—some of the different plausible strategies that may be used in communication policies directed to promote better evaluations and positioning of those products. Specifically, this aim was pursued through an online non-hypothetical auction, for evaluating the price premium that consumers were willing to pay for Summer Mountain Cheese (SMC) over Winter Mountain Cheese (WMC), depending on the information provided.

Among the most important results, the difference in willingness to pay for SMC and WMC is qualified by the type of information received, whereas the absence of a significant premium price in the control condition is not surprising. Unexpectedly, participants in the *info-ANW* condition also did not show a significantly higher WTP for SMC compared to WMC. Therefore, it seems that the message founded upon sensorial characteristics and anticipated taste of the cheese was more effective that the message relying on the concept of animal welfare.

The experimental results, which showed that taste information was more effective in increasing the consumer’s WTP for the summer cheese, corroborated the evidence from Napolitano et al. [77] and Gross et al. [78] that introduced a tasting session during the lab sessions and found that consumers were willing to pay more for animal-friendly products only if they tasted good.

On the other hand, it was not expected that consumers were indifferent to the message about animal welfare. This is somewhat surprising considering that a recent meta-analysis on this topic indicated a positive price premium for animal-friendly products [79]. In fact, in most cases, consumers are prone to pay more for animal-friendly products given the provision of positive information about animal welfare [80,81,82,83,84]. However, some studies suggested that rational information on animal welfare might have different results. 

For instance, when negative outcomes of animal welfare enhancement were provided alone, consumers’ WTP for animal welfare decreased, while, when they were provided jointly with positive information, consumers were willing to pay the same price or less for the animal-friendly product compared to the conventional one [80,85]. Furthermore, Elbakidze and Nayga [86] found that explaining the animal-care content of cheese and ice-cream to American consumers decreased their willingness to pay for the products. Overall, research on this topic seems to provide mixed results and would, thus, be worthy of further investigation.

However, the significant and positive interaction found between message and logo—which is in line with expectations—appears to be the most relevant and innovative result of the current study. After inspecting the mean difference between the WTP for SMC and WMC across the nine conditions, it emerged that participants who received the *info-TAS* message and saw the *free-range* logo expressed a significant premium price of 1.43 Euros. This group of participants was the only one to express a significant premium price. This suggests that the influence of the *info-TAS* message was reinforced by the *free-range* logo, but not by the grass-fed logo. 

One possible interpretation of this interesting result could be that the condition info-TAS*logo-FRE is the only one, among the different combinations of messages and logos, to show a clear combination of egoistic and altruistic motives (see for example [87,88,89,90]. The *info-TAS* message influences the individuals’ egoistic/hedonistic motivation, in that experimental cell, this message may have been reinforced and complemented by the logo on *free-range*, which elicits altruistic motivation instead and perhaps reassures participants from an ethical point of view. 

From another perspective, the free-range logo taps more into moral and social emotions (e.g., guilt and shame—see Tangney et al. [91] for an overview); it is reasonable to think that it could have a positive and reassuring effect on participants. The link of the grass-fed logo with ethical profiles, as well as with moral and social emotion, is perhaps less effective: this could explain the absence of a significant interactive effect of *info-TAS* by grass-fed on the participants’ price premium. This idea of a multiplicative effect based on complementarity between hedonistic and ethical communication is now a merely speculative post-hoc interpretation of the results; nevertheless, it could be further explored by future research to reveal relevant and novel insights.

## 6. Conclusions

To ensure the persistence of mountain livestock farming, an adequate remuneration of high-quality dairy products is fundamental. In this sense, communication strategies aimed at enhancing mountain products peculiarities play a key role in unlocking the market potential. The present research expands the literature on consumers’ willingness to pay for mountain foods by using an online real auction experiment aimed at evaluating the price premium that consumers are willing to pay for summer over winter mountain cheese, depending on the information provided. The results show an overall small premium price given to the higher quality summer cheese; this could be partially due to a generally low degree of consumers’ knowledge about mountain dairy farming.

With reference to communication strategies, the results provide evidence about the effectiveness of the rational messages founded upon sensorial characteristics and the anticipated taste of the cheese. Adding a logo, instead, had no main effect on the price premium that participants were willing to pay. This may be due to the fact that logos and claims, having a lower information content, are more indicated for leading the choice of consumers with higher levels of awareness.

Some practical implications can be drawn from the results. From a marketing perspective, the findings suggest that communication strategies should be focused on informative messages that are able to increase the consumer knowledge, awareness, and—as a consequence—their willingness to pay for mountain products. This is clearly crucial, as the content of the message and the way it is presented to consumers can affect the purchase intention and WTP. In this respect, a recent study on the labelling of food safety attributes highlighted that food labels characterized by unsubstantiated claims could determine higher premiums, compared to labels that offer factual information [92]. 

From a policy standpoint, specific communication campaigns aimed to raise awareness about the benefits connected to mountain livestock farming are highly recommended. Moreover, policy makers should also develop targeted dissemination events, involving mountain dairy chain actors, in order to increase the average knowledge about mountain farming practices. Finally, the suggested possible multiplicative effect between communication elements belonging to the hedonistic and ethical domains—if supported by further empirical evidence—might be a viable avenue for increasing the effectiveness of communication strategies and policies.

Certain limitations must be acknowledged. First, only one experimental treatment was significant and had a small effect size. This suggests that the premium price due to the combination of message on taste and the free-range logo need to be confirmed by further studies to exclude a random origin. Secondly, despite the use of the BDM online auction representing a novelty in food marketing research, the experimental design did not test the reliability of this method compared to more popular auction methods or to exclude any potential bias due to the way the data were collected. 

Furthermore, the research was conducted only in Italy and during the SarsCoV2 pandemic; thus, some doubt might be raised regarding the external validity of the results and the effect of the pandemic on consumers’ preferences for food. However, Italy could represent an adequate good case study per se, due to the localization of the production of the cheese considered in the analysis. 

Regarding the SarsCoV2 pandemic, the data collection started at the end of the first Italian lockdown phase, when social restriction and contagion rates were the lowest in that period. Third, no measure of previous knowledge regarding the cheese products was implemented in the experiment. Future research should focus on determining how previous knowledge interacts with consumer valuation behavior [93].

Finally, the effect size and methodological issues might suggest the best direction for future research. Specifically, there is a need to confirm the interpretation of the results, which means expanding the exploration of the effect of the combined hedonic and pro-social stimulus on consumers’ willingness to pay for enhanced food produce. A comparative analysis between well-known experimental auction methods and online BDM auction would be an important contribution to the scientific community.

## Figures and Tables

**Table 1:** Descriptive statistics of the sample (*n* = 849).

Characteristics	Frequency	% Sample	% Italy
*Gender*			
Male	426	50.18	48.36
Female	423	49.82	51.64
*Age*			
18–24	66	7.77	6.76
25–34	148	17.43	14.57
35–44	188	22.14	19.27
45–54	167	19.67	18.41
55–64	142	16.73	15.41
65–74	124	14.61	12.87
75+	14	1.65	12.71
*Education*			
Below Upper Secondary	77	9.07	40.10
Upper Secondary	416	49.00	42.30
Tertiary	356	41.94	17.60
*Household members*			
1	80	9.47	13.00
2	237	28.05	22.50
3	221	26.15	24.80
4	226	26.75	26.90
5+	81	9.58	12.84
*Geographical location*			
North-East	157	18.50	19.44
North-West	225	26.50	26.40
Central	170	20.02	19.84
Southern and Islands	297	34.98	34.42

**Table 2:** Descriptive statistics of the bids on the two products.

	Bids Including Zero				Bids Excluding Zero			
	*n*	Mean WTP	SD	Skewness	*n*	Mean WTP	SD	Skewness
Winter Cheese	849	2.80	4.42	5.76	600	3.97	4.80	5.71
Summer Cheese	849	2.91	4.89	5.97	602	4.11	5.37	5.75

WTP: Willingness To Pay.

**Table 3:** The mean and standard deviations of the bids on the two products across the experimental groups.

		Bids on Winter Cheese			Bids on Summer Cheese		
Info	Logo	Mean	SD	*N*	Mean	SD	*N*
No info	No Logo	2.40	2.80	91	2.30	2.99	91
	Free-range	2.91	3.62	96	2.50	3.27	96
	Grass-fed	3.14	5.68	93	3.16	6.60	93
	Total	2.82	4.21	280	2.65	4.58	280
Taste	No Logo	2.54	3.33	96	2.65	3.06	96
	Free-range	2.33	3.74	96	3.75	7.41	96
	Grass-fed	3.45	4.75	92	3.43	4.36	92
	Total	2.76	3.99	284	3.28	5.28	284
Animal Welfare	No Logo	3.05	6.50	93	3.43	6.75	93
	Free-range	2.38	3.18	96	2.31	3.25	96
	Grass-fed	3.09	4.85	95	2.72	3.61	95
	Total	2.84	5.00	284	2.81	4.78	284
Total	No Logo	2.66	4.51	280	2.79	4.62	280
	Free-range	2.54	3.52	288	2.85	5.06	288
	Grass-fed	3.23	5.09	280	3.10	5.00	280
	Total	2.81	4.42	848	2.92	4.89	848

**Table 4:** The mean difference between the WTP for SMC and WMC across message and logo conditions.

		Δ WTP Mean (SMC—WMC)	SD	t	*p*
No Info	No Logo	−0.103	1.30	0.756	0.452
	Free-range	−0.411	2.27	1.77	0.08
	Grass-fed	0.019	2.15	0.084	0.934
Taste	No Logo	0.105	2.01	0.509	0.612
	Free-range	1.429	6.61	2.119	0.037
	Grass-fed	−0.010	1.74	0.059	0.953
Animal Welfare	No Logo	0.381	4.60	0.80	0.426
	Free-range	−0.069	2.48	0.272	0.786
	Grass-fed	−0.372	2.36	1.541	0.127

Δ: Change of Willingness to Pay; SMC: Summer Mountain Cheese; and WMC: Winter Mountain Cheese.

**Figure A1:**
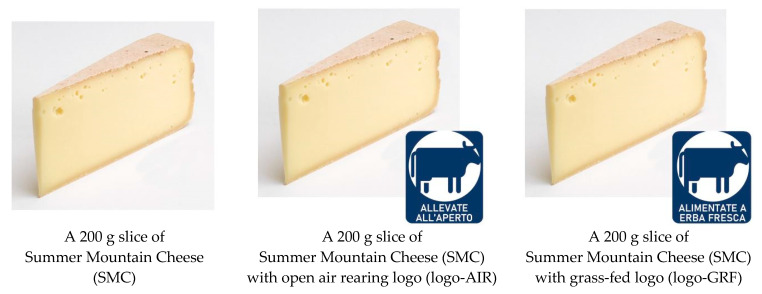
The summer and winter mountain cheeses auctioned.

## Data Availability

Data will be provided upon reasonable request to the contact author.

## Appendix A

Informative Rational Message used in the study:

*(1) Taste*

What are winter cheeses and summer cheeses?
Cheeses produced in mountain areas are mainly divided in “winter” and “summer” cheeses.
“Winter” cheeses are produced between October and April, whereas “summer” cheeses are produced from May to the end of September.
In any case, both types of cheese are aged and then sold during the rest of the year. In this way, the so-called “deseasonalization of production” is realized and mountain cheese is always present on the market.
Winter cheese is made from the milk of cows that are fed in the barn with local dry fodder and feed. On the contrary, summer cheese derives from the milk of cows fed with fresh local fodders, which give a unique and very rich aromatic and sensorial profile. Summer cheese is generally considered to have a better taste.

*(2) Animal Welfare*

What are winter cheeses and summer cheeses?
Cheeses produced in mountain areas are mainly divided in “winter” and “summer” cheeses.
“Winter” cheeses are produced between October and April, whereas “summer” cheeses are produced from May to the end of September.
In any case, both types of cheese are aged and then sold during the rest of the year. In this way, the so-called “deseasonalization of production” is realized and mountain cheese is always present on the market.
Winter cheese is made from the milk of cows that are fed in the barn with local dry fodder and feed. On the contrary, summer cheese comes from the milk of cows fed with fresh local fodder, which in most cases live free in the open air. This is generally believed to promote cow welfare.

## References

[1] Alpine Convention Demographic Changes in the Alps—Report on the State of the Alps.

[2] Endrizzi I., Cliceri D., Menghi L., Aprea E., Gasperi F. (2021). Does the ‘Mountain Pasture Product’ claim affect local cheese acceptability?. Foods.

[3] Sturaro E., Marchiori E., Cocca G., Penasa M., Ramanzin M., Bittante G. (2013). Dairy systems in mountainous areas: Farm animal biodiversity, milk production and destination, and land use. Livest. Sci..

[4] Ferrario E., Price M. (2014). Should I stay or should I go? Alpine brain drain and brain gain: The reasons behind the choices of young mountain people. J. Alpine Res. Rev. Géogr. Alpine.

[5] Ramanzin M., Salvador S., Sturaro E., Bovolenta S. (2014). Livestock Farming Systems in the Eastern Italian Alps: Ecosystem Services and Product Quality. Opt. Méditerr..

[6] Faccioni G., Sturaro E., Ramanzin M., Bernués A. (2019). Socio-economic valuation of abandonment and intensification of Alpine agroecosystems and associated ecosystem services. Land Use Policy.

[7] Santini F., Guri F., Gomez y Paloma S. (2013). Labelling of Agricultural and Food Products of Mountain Farming.

[8] Riddlesden D., Singleton A.D. (2014). Broadband speed equity: A new digital divide?. Appl. Geogr..

[9] Demartini E., Gaviglio A., Bertoni D. (2015). Integrating agricultural sustainability into policy planning: A geo-referenced framework based on Rough Set theory. Environ. Sci. Policy.

[10] Dehnen-Schmutz K., Foster G.L., Owen L., Persello S. (2016). Exploring the role of smartphone technology for citizen science in agriculture. Agron. Sustain. Dev..

[11] Marescotti M.E., Demartini E., Filippini R., Gaviglio A. (2021). Smart farming in mountain areas: Investigating livestock farmers’ technophobia and technophilia and their perception of innovation. J. R. Stud..

[12] Filippini R., Marescotti M.E., Demartini E., Gaviglio A. (2020). Social Networks as Drivers for Technology Adoption: A Study from a Rural Mountain Area in Italy. Sustainability.

[13] Gutiérrez-Peña R., Fernández-Cabanás V.M., Mena Y., Delgado-Pertíñez M. (2018). Fatty acid profile and vitamins A and E contents of milk in goat farms under Mediterranean wood pastures as affected by grazing conditions and seasons. J. Food Compos. Anal..

[14] Bertoni D., Cavicchioli D., Pretolani R., Olper A. (2011). Agri-environmental measures adoption: New evidence from Lombardy region. The Common Agricultural Policy after the Fischler Reform: National Implementations, Impact Assessment and the Agenda for Future Reforms.

[15] MacDonald D., Crabtree J.R., Wiesinger G., Dax T., Stamou N., Fleury P., Gutierrez Lazpita J., Gibon A. (2000). Agricultural abandonment in mountain areas of Europe: Environmental consequences and policy response. J. Environ. Manag..

[16] Dax T. (2001). Endogenous Development in Austria’s Mountain Regions. Mt. Res. Dev..

[17] Mazzocchi C., Sali G. (2018). Assessing the value of pastoral farming in the Alps using choice experiments: Evidence for public policies and management. J. Environ. Plan. Manag..

[18] Cavicchioli D., Bertoni D., Tesser F., Frisio D.G. (2015). What factors encourage intrafamily farm succession in mountain areas? Evidence from an Alpine valley in Italy. Mt. Res. Dev..

[19] Manzo A., Panseri S., Bertoni D., Giorgi A. (2015). Economic and qualitative traits of Italian Alps saffron. J. Mt. Sci..

[20] Mazzocchi C., Sali G. (2016). Sustainability and competitiveness of agriculture in mountain areas: A Willingness to Pay (WTP) approach. Sustainability.

[21] Demartini E., Gaviglio A., Pirani A. (2017). Farmers’ motivation and perceived effects of participating in short food supply chains: Evidence from a North Italian survey. Agric. Econ..

[22] Feldmann C., Hamm U. (2015). Consumers’ perceptions and preferences for local food: A review. Food Qual. Prefer..

[23] Asioli D., Aschemann-Witzel J., Caputo V., Vecchio R., Annunziata A., Næs T., Varela P. (2017). Making sense of the “clean label” trends: A review of consumer food choice behavior and discussion of industry implications. Food Res. Int..

[24] Rana J., Paul J. (2017). Consumer behavior and purchase intention for organic food: A review and research agenda. J. Retail. Consum. Serv..

[25] Marescotti M.E., Caputo V., DeMartini E., Gaviglio A. (2020). Consumer preferences for wild game cured meat label: Do attitudes towards animal welfare matter?. Int. Food Agribus. Manag. Rev..

[26] Stanton C., Mills S., Ryan A., Di Gioia D., Ross R. (2021). Influence of pasture feeding on milk and meat products in terms of human health and product quality. Ir. J. Agric. Food Res..

[27] Alothman M., Hogan S.A., Hennessy D., Dillon P., Kilcawley K.N., O’Donovan M., Tobin J., Fenelon M.A., O’Callaghan T.F. (2019). The “Grass-Fed” Milk Story: Understanding the Impact of Pasture Feeding on the Composition and Quality of Bovine Milk. Foods.

[28] Lombardi G., Peira G., Cortese D. (2019). The Supply Chains of Cow Grass-Fed Milk.

[29] Provenza F.D., Kronberg S.L., Gregorini P. (2019). Is Grassfed Meat and Dairy Better for Human and Environmental Health?. Front. Nutr..

[30] Savoini G., Agazzi A., Invernizzi G., Cattaneo D., Pinotti L., Baldi A. (2010). Polyunsaturated fatty acids and choline in dairy goats nutrition: Production and health benefits. Small Rumin. Res..

[31] Delgado-Pertíñez M., Gutiérrez-Peña R., Mena Y., Fernández-Cabanás V.M., Laberye D. (2013). Milk production, fatty ac-id composition and vitamin E content of Payoya goats according to grazing level in summer on Mediterranean shrublands. Small Rumin. Res..

[32] Capuano E., van der Veer G., Boerrigter-Eenling R., Elgersma A., Rademaker J.L., Sterian A., van Ruth S.M. (2014). Verification of fresh grass feeding, pasture grazing and organic farming by cows farm milk fatty acid profile. Food Chem..

[33] Valdivielso I., Albisu M., de Renobales M., Barron L.J.R. (2016). Changes in the volatile composition and sensory properties of cheeses made with milk from commercial sheep flocks managed indoors, part-time grazing in valley, and extensive mountain grazing. Int. Dairy J..

[34] Dewhurst R., Shingfield K., Lee M., Scollan N. (2006). Increasing the concentrations of beneficial polyunsaturated fatty acids in milk produced by dairy cows in high-forage systems. Anim. Feed Sci. Technol..

[35] La Terra S., Marino V.M., Manenti M., Licitra G., Carpino S. (2010). Increasing pasture intakes enhances polyunsaturated fatty acids and lipophilic antioxidants in plasma and milk of dairy cows fed total mix ration. Dairy Sci. Technol..

[36] Coppa M., Martin B., Pradel P., Leotta B., Priolo A., Vasta V. (2011). Effect of a Hay-Based Diet or Different Upland Grazing Systems on Milk Volatile Compounds. J. Agric. Food Chem..

[37] Shingfield K., Bonnet M., Scollan N. (2013). Recent developments in altering the fatty acid composition of ruminant-derived foods. Animal.

[38] Savoini G., Farina G., Dell’Orto V., Cattaneo D. (2016). Through ruminant nutrition to human health: Role of fattyacids. Adv. Anim. Biosci..

[39] Calder P.C. (2013). Omega-3 e polyunsaturated fatty acids and inflammatory processes: Nutrition or pharmacology?. Br. J. Clin. Pharmacol..

[40] WHO (2008). Interim Summary of Conclusions and Dietary Recommendations on Total Fatfatty Acids from the Joint FAO/WHO Expert Consultation on Fats and Fatty Acids in Human Nutrition.

[41] Buchin S., Martin B., Dupont D., Bornard A., Achilleos C. (1999). Influence of the composition of Alpine highland pasture on the chemical, rheological and sensory properties of cheese. J. Dairy Res..

[42] Bugaud C., Buchin S., Coulon J.-B., Dupont D. (2001). Influence of the nature of alpine pastures on plasmin activity, fatty acid and volatile compound composition of milk. Le Lait.

[43] Carpino S., Horne J., Melilli C., Licitra G., Barbano D.M., Van Soest P.J. (2004). Contribution of native pasture to the sensory properties of Ragusano cheese. J. Dairy Sci..

[44] Coulon J.-B., Martin B., Pirisi A. (2004). Relationships between ruminant management and sensory characteristics of cheeses: A review. Le Lait.

[45] Abilleira E., Schlichtherle-Cerny H., Virto M., de Renobales M., Barron L.J.R. (2010). Volatile composition and aroma-active compounds of farmhouse Idiazabal cheese made in winter and spring. Int. Dairy J..

[46] Zuliani A., Esbjerg L., Grunert K.G., Bovolenta S. (2018). Animal Welfare and Mountain Products from Traditional Dairy Farms: How Do Consumers Perceive Complexity?. Animals.

[47] Spigarelli C., Zuliani A., Battini M., Mattiello S., Bovolenta S. (2020). Welfare assessment on pasture: A review on animal-based measures for ruminants. Animals.

[48] Van den Pol-van Dasselaar A., Goliński P., Hennessy D., Huyghe C., Parente G., Peyraud J.-L. (2014). Évaluation des fonctions des prairies par les acteurseuropéens. Fourrages.

[49] Sanjuan A.I., Khliji S. (2016). Urban consumers’ response to the EU food mountain labelling: An empirical application in Southern Europe. New Medit.

[50] Nam K., Lim H., Ahn B.-I. (2020). Analysis of Consumer Preference for Milk Produced through Sustainable Farming: The Case of Mountainous Dairy Farming. Sustainability.

[51] Mazzocchi C., Orsi L., Sali G. (2021). Consumers’ Attitudes for Sustainable Mountain Cheese. Sustainability.

[52] Ferrari L., Cavaliere A., De Marchi E., Banterle A. (2019). Can nudging improve the environmental impact of food supply chain? A systematic review. Trends Food Sci. Technol..

[53] Cadario R., Chandon P. (2020). Which Healthy Eating Nudges Work Best? A Meta-Analysis of Field Experiments. Mark. Sci..

[54] Verneau F., La Barbera F., Kolle S., Amato M., Del Giudice T., Grunert K. (2016). The effect of communication and implicit associations on consuming insects: An experiment in Denmark and Italy. Appetite.

[55] Demartini E., De Marchi E., Cavaliere A., Mattavelli S., Gaviglio A., Banterle A., Richetin J., Perugini M. (2019). Changing attitudes towards healty food via self-association or nutritional information: What works best?. Appetite.

[56] Murphy J.J., Allen P.G., Stevens T.H., Weatherhead D. (2005). A meta-analysis of hypothetical bias in stated preference valuation. Environ. Resour. Econ..

[57] Harrison G.W., Rutström E.E. (2008). Chapter 81 Experimental Evidence on the Existence of Hypothetical Bias in Value Elicitation Methods. Handbook of Experimental Economics Results.

[58] Verneau F., La Barbera F., Del Giudice T. (2016). The Role of Implicit Associations in the Hypothetical Bias. J. Consum. Aff..

[59] Canavari M., Drichoutis A.C., Lusk J.L., Nayga R.M. (2019). How to run an experimental auction: A review of recent advances. Eur. Rev. Agric. Econ..

[60] Menta G., Venuti M. (2014). Esempi di sostenibilità di alcune aziende zootecniche di montagna in cui si allevano bovine di razza Pezzata Rossa Italiana. S. Bovolenta e E. Sturaro (a cura di) I formaggi protagonisti della zootecnia alpina. Quad. SoZooAlp (SoZooAlp Trento).

[61] Becker G.M., DeGroot M.H., Marschak J. (1964). Measuring utility by a single-response sequential method. Syst. Res. Behav. Sci..

[62] Lemken D., Knigge M., Meyerding S., Spiller A. (2017). The Value of Environmental and Health Claims on New Legume Products: A Non-Hypothetical Online Auction. Sustainability.

[63] Vickrey W. (1961). Counterspeculation, auctions, and competitive sealed tenders. J. Financ..

[64] Kuijken B., Gemser G., Wijnberg N.M. (2017). Categorization and willingness to pay for new products: The role of category cues as value anchors. J. Prod. Innov. Manag..

[65] Onderstal S. (2020). Premium auctions in the field. Econ. Des..

[66] Ginon E., Chabanet C., Combris P., Issanchou S. (2014). Are decisions in a real choice experiment consistent with reservation prices elicited with BDM ‘auction’? The case of French baguettes. Food Qual. Prefer..

[67] Ginon E., Lohéac Y., Martin C., Combris P., Issanchou S. (2009). Effect of fibre information on consumer willingness to pay for French baguettes. Food Qual. Prefer..

[68] La Barbera F. (2015). Framing the EU as common project vs. common heritage: Effects on attitudes towards the EU deepening and widening. J. Soc. Psychol..

[69] La Barbera F., Ajzen I. (2020). Understanding support for European integration across generations: A study guided by the theory of planned behavior. Eur. J. Psychol..

[70] ISTAT (2012). 15 Censimento Generale Della Popolazione e Delle Abitazioni. https://www.istat.it/it/files/2012/12/volume_popolazione-legale_XV_censimento_popolazione.pdf.

[71] OECD (2016). Education at a Glance 2016: OECD Indicators.

[72] Capecchi S., Amato M., Sodano V., Verneau F. (2019). Understanding beliefs and concerns towards palm oil: Empirical evidence and policy implications. Food Policy.

[73] Demartini E., Vecchiato D., Tempesta T., Gaviglio A., Viganò R. (2018). Consumer preferences for red deer meat: A discrete choice analysis considering attitudes towards wild game meat and hunting. Meat Sci..

[74] Nocella G., Hubbard L., Scarpa R. (2010). Farm animal welfare, consumer willingness to pay, and trust: Results of a crossnational survey. Appl. Econ. Perspect. Policy.

[75] Iweala S., Lemken D. Utilizing the warm glow of giving to nudge the consumption of food items with ethical claims–an experimental online auction. Proceedings of the 2019 Sixth International Conference African Association of Agricultural Economists (AAAE).

[76] Amato M., Ballco P., López-Galán B., De Magistris T., Verneau F. (2017). Exploring consumers’ perception and willingness to pay for “Non-Added Sulphite” wines through experimental auctions: A case study in Italy and Spain. Wine Econ. Policy.

[77] Napolitano F., Pacelli C., Girolami A., Braghieri A. (2008). Effect of Information About Animal Welfare on Consumer Willingness to Pay for Yogurt. J. Dairy Sci..

[78] Gross S., Waldrop M.E., Roosen J. (2020). How does animal welfare taste? Combining sensory and choice experiments to evaluate willingness to pay for animal welfare pork. Food Qual. Prefer..

[79] Clark B., Stewart G.B., Panzone L.A., Kyriazakis I., Frewer L.J. (2017). Citizens, consumers and farm animal welfare: A meta-analysis of willingness-to-pay studies. Food Policy.

[80] Akaichi F., Nayga R.M., Gil J.M. (2012). Assessing Consumers’ Willingness to Pay for Different Units of Organic Milk: Evidence from Multiunit Auctions. Can. J. Agric. Econ. Can. D’agroecon..

[81] Risius A., Hamm U. (2017). The effect of information on beef husbandry systems on consumers’ preferences and willingness to pay. Meat Sci..

[82] Ankamah-Yeboah I., Jacobsen J.B., Olsen S.B., Nielsen M., Nielsen R. (2019). The impact of animal welfare and environmental information on the choice of organic fish: An empirical investigation of German trout consumers. Mar. Resour. Econ..

[83] Ochs D., Wolf C.A., Widmar N.O., Bir C., Lai J. (2019). Hen housing system information effects on U.S. egg demand. Food Policy.

[84] Scozzafava G., Gerini F., Boncinelli F., Contini C., Marone E., Casini L. (2019). Organic milk preference: Is it a matter of information?. Appetite.

[85] Cao Y., Cranfield J., Chen C., Widowski T. (2020). Heterogeneous informational and attitudinal impacts on consumer preferences for eggs from welfare enhanced cage systems. Food Policy.

[86] Elbakidze L., Nayga R.M. (2012). The effects of information on willingness to pay for animal welfare in dairy production: Application of nonhypothetical valuation mechanisms. J. Dairy Sci..

[87] Birch D., Memery J., Kanakaratne M.D.S. (2018). The mindful consumer: Balancing egoistic and altruistic motivations to purchase local food. J. Retail. Consum. Serv..

[88] Pohjanheimo T., Paasovaara R., Luomala H., Sandell M. (2010). Food choice motives and bread liking of consumers embracing hedonistic and traditional values. Appetite.

[89] Shaw D., Shiu E. (2002). The role of ethical obligation and self-identity in ethical consumer choice. Int. J. Consum. Stud..

[90] O’Shaughnessy J., Jackson O’Shaughnessy N. (2002). Marketing, the consumer society and hedonism. Eur. J. Mark..

[91] Tangney J.P., Stuewig J., Mashek D.J. (2007). Moral Emotions and Moral Behavior. Annu. Rev. Psychol..

[92] Britwum K., Yiannaka A. (2019). Labeling food safety attributes: To inform or not to inform?. Agric. Food Econ..

[93] Meerza S.I.A., Gustafson C.R. (2019). Does prior knowledge of food fraud affect consumer behavior? Evidence from an incentivized economic experiment. PLoS ONE.

